# Emerging from the COVID-19 pandemic: the numbers and lessons that will stay with us forever

**DOI:** 10.31744/einstein_journal/2021ED6207

**Published:** 2021-02-19

**Authors:** Luiza Helena Degani-Costa, Fabiana Rolla, Raphael Augusto Gomes Oliveira, Guilherme de Paula Pinto Schettino, Ricardo Luiz Cordioli, Fábio Barlem Hohmann, Niklas Söderberg Campos, Roger Monteiro Alencar, Leonardo José Rolim Ferraz, Felipe Maia de Toledo Piza

**Affiliations:** 1 Hospital Israelita Albert EinsteinSão PauloBrazil Hospital Israelita Albert Einstein , São Paulo , SP, Brazil .; 2 Hospital Municipal Dr. Moysés DeutschSão PauloSPBrazil Hospital Municipal Dr. Moysés Deutsch , São Paulo , SP , Brazil .

In Brazil, the first patient with coronavirus 2019 disease (COVID-19) was diagnosed on February 26, 2020. He lived in the city of São Paulo (SP), Brazil, and had recently returned from a trip to Europe. Over the next couple of weeks, most of the new cases of COVID-19 had an identifiable epidemiological risk factor – either having travelled abroad or been in contact with a patient known to have severe acute respiratory syndrome coronavirus 2 (SARS-CoV-2) infection. However, as initial containment measures failed, the epidemic that had started in the high-income brackets quickly spread to the whole community, hitting people of low-income brackets in a particularly hard manner. ^( 1 )^ As of August 31 ^st^ , 2020, a total of 257,778 cases had been confirmed in the city of São Paulo, and 11,400 deaths had been attributed to COVID-19 (https://www.seade.gov.br/coronavirus/).

In this context, the public and private health systems in São Paulo were forced to make adjustments, often coming together to allow rational and efficient use of limited medical resources. Several public health measures put into place in response to the SARS-CoV-2 pandemic, including city-level quarantine and mandatory widespread use of cloth or surgical masks later on. However, establishing temporary field hospital facilities and appointing selected public hospitals as reference centers for treatment of COVID-19 were the cornerstone of the public healthcare policy.

*Hospital Municipal Dr. Moysés Deustch* ( *M’Boi Mirim –* HMMD) was one of these hospitals. Managed by a large philanthropic medical organization, *Hospital Israelita Albert Einstein* (HIAE), the HMMD is a unique community hospital in São Paulo, located in one of the largest underdeveloped suburbs in the city and responsible for the care of over one million people. It also serves as a teaching hospital for medical students and several residency programs. During the COVID-19 pandemic, the mission of the organization and its preparedness plan led the hospital to become a major center COVID-19 center in the country.

Since the beginning of the pandemic, this originally 210-bed hospital has received 3,237 patients with suspected or confirmed COVID-19. When analyzing the first 616 patients admitted, it was observed our epidemiology was similar to what has been described at international levels, ^( 2 )^ except for a relatively younger population: 57.4% were male, median age of 55 years, range of zero to 95 years. Most common comorbidities were hypertension (53.6%), diabetes (35.7%) and obesity (24%). Although less than 10% of study sample was known to have chronic respiratory diseases, one quarter of the patients were former or current smokers. The median duration of symptoms before hospital admission was 7 days and most patients presented with fever (72.2%), cough (79.6%) and dyspnea (78.3%). Myalgia and fatigue were also common complaints.

Upon presentation, almost 70% of patients required some oxygen supplementation or had lung infiltrates taking up more than 50% of parenchyma, and were therefore considered as severe cases. While time from onset of symptoms was not different between severe or mild disease patients, those with severe disease were older, had more comorbidities (especially diabetes, hypertension and other chronic cardiovascular diseases), and presented with lower arterial oxygen partial pressure to fractional inspired oxygen ratio (PaO _2_ /FiO _2_ ). The initial oxygen supplementation was provided by means of nasal cannula (60.4%) or non-rebreather masks (17.8%) to most severe cases, but 9.6% required immediate invasive mechanical ventilation.

Overall, we managed to obtain clinical outcomes similar to those reported in the United States and European countries, ^( 3 )^ with an in-hospital mortality of 24.5%. As expected, those with mild disease had shorter hospital stay (median 3 *versus* 7 days), were discharged home sooner (64.2% *versus* 37%) and had lower in-hospital mortality (6.6% *versus* 32.8%) than patients who presented with severe disease upon admission. In addition, patients with severe disease more often required intensive care (39.8% *versus* 8.1%), mechanical ventilation (39.9% *versus* 9.8%), vasopressors (30.9% *versus* 8.1%) and neuromuscular blockade (27% *versus* 4.9%) at some point during hospital stay. Furthermore, the incidence of acute kidney injury was higher among patients with severe disease (35% *versus* 12.5%), as well as the need for renal replacement therapy (17.9% *versus* 2.5%). It is important to stress that all these numbers reflect the reality before the results of the RECOVERY trial, ^( 4 )^ when steroids were given only to a small fraction of patients enrolled in clinical studies.

How did a Secondary Care community hospital, which historically struggled to make ends meet, achieve results comparable to American and European centers? Distinguishing crisis from disaster is the first step to create a sense of urgency. Crisis requires decision and preparedness. Disaster, in contrast, is an unpredictable event beyond any control. By relying on international experience and frequently analyzing its own dataset, HMMD was able to anticipate and prepare the organization for the crisis to come, learning and sharing experiences with other medical institutions in the country and abroad.

Rational and cost-effective allocation of resources was fundamental for an assertive response to the coming crisis. ^( 5 )^ As depicted by the statistics presented here, both human and technological resources had to be rapidly increased to meet the rising demand of complex and critical patients. In an unprecedented level of collaboration with HIAE and the private industry, HMMD received 190 mechanical ventilators, 60 multiparametric monitors and 30 hemodialysis machines in a matter of weeks. Furthermore, several areas of the hospital (such as medical and surgical wards, operating rooms and outpatient clinics) were quickly adapted to receive critical patients. Impressively, a new annex hospital with the capacity for one hundred patients was built in only 23 days. The net result was a blitzscaling process through which the medium-size secondary care community hospital was essentially transmuted into a large reference center with a capacity for 524 patients and 220 critical care beds, within less than a month.

When it comes to human resources, 1,080 new positions were open for physicians and allied health professionals. Continuous training and communication using simulation and videoconferencing tools were fundamental to keep staff engaged and updated about the management of COVID-19. It was remarkable to see that the crisis had brought back a deep sense of purpose and teamwork for many healthcare professionals. Purpose, not salary, was what drove colleagues into coming to work with us. It was a calling to make a difference, to create a legacy regardless of the extra shifts and exhaustion. Interestingly (and fortunately), we managed to convey this same sense of purpose to our medical students and residents, who kept on with their training and contributed significantly to care of patients.

Currently, as infection rates decrease and we emerge from the COVID-19 pandemic, it is only natural that we look back and meditate about it. As of the day of this writing, 554 lives were lost at our hospital since March. They were someone else’s parents, grandparents, sons and daughters. But while their unrepairable loss has prompted repeated revisions of our institutional processes and guidelines, and 2,181 patients who were successfully treated and discharged home are the living proof of the three most important lessons of this pandemic: first, we can operate miracles when the private and public sectors come together with the people’s best interest at heart; second, collaborative work is the best way to build up knowledge and it saves lives; and third, from an individual standpoint, we may never experience such tremendous personal growth again in our lifetime.
