# Supplementary material for: Emerging from the COVID-19 pandemic: the numbers and lessons that will stay with us forever
Source: Einstein (Sao Paulo). 2021 Feb 19;19:eED6207. doi: 10.31744/einstein_journal/2021ED6207 (PMC7898589; doi:10.31744/einstein_journal/2021ED6207)
Supplement: Translation

# Translation: Emergindo da pandemia de COVID-19: os números e as lições que permanecerão conosco para sempre

**Authors:** Luiza Helena Degani-Costa, Fabiana Rolla, Raphael Augusto Gomes Oliveira, Guilherme de Paula Pinto Schettino, Ricardo Luiz Cordioli, Fábio Barlem Hohmann, Niklas Söderberg Campos, Roger Monteiro Alencar, Leonardo José Rolim Ferraz, Felipe Maia de Toledo Piza

**Affiliations:** 1 Brasil Hospital Israelita Albert Einstein, São Paulo, SP, Brasil .; 2 Brasil Hospital Municipal Dr. Moysés Deutsch, São Paulo, SP, Brasil .

No Brasil, o primeiro paciente com a doença do coronavírus 2019 (COVID-19) foi diagnosticado em 26 de fevereiro de 2020. Morava em São Paulo (SP) e retornara recentemente de uma viagem da Europa. Ao longo das semanas seguintes, a maioria dos novos casos de COVID-19 tinha um fator de risco epidemiológico identificável – ter viajado ao exterior ou tido contato com um paciente que sabidamente estava com o coronavírus da síndrome respiratória aguda grave 2 (SARS-CoV-2). No entanto, como as medidas iniciais de contenção falharam, a epidemia, que começou nas classes mais altas, rapidamente se espalhou para toda a comunidade, atingindo de forma muito dura as pessoas das classes mais desfavorecidas. ^( 1 )^ Desde 31 de agosto de 2020, 257.778 casos já foram confirmados na cidade de São Paulo, e 11.400 mortes foram atribuídas à COVID-19 (https://www.seade.gov.br/coronavirus/).

Sistemas de saúde pública e privada em São Paulo foram forçados a fazer ajustes, frequentemente juntos, para permitir o uso racional e eficiente de recursos médicos limitados. Várias medidas de saúde pública implementadas em resposta à pandemia de SARS-CoV-2, incluindo uma quarentena no município e, logo depois, o uso obrigatório de máscaras de tecido ou cirúrgicas. Porém, hospitais de campanha temporários e a escolha de hospitais públicos selecionados para se tornarem centros de referência para o tratamento da COVID-19 foram pilares da política de saúde pública.

O Hospital Municipal Dr. Moysés Deutsch (M’Boi Mirim – HMMD) foi um desses hospitais. Sob a gerência de uma grande instituição médica filantrópica, o Hospital Israelita Albert Einstein (HIAE), o HMMD é um hospital de comunidade singular em São Paulo, localizado em um dos maiores subúrbios subdesenvolvidos da cidade e responsável pelo cuidado de mais de 1 milhão de pessoas. É também um hospital-escola para estudantes de medicina e vários programas de residência médica. Durante a pandemia de COVID-19, a missão e a preparação da instituição tornaram o hospital em um importante centro de COVID-19 no país.

Desde o início da pandemia, este hospital, que, inicialmente, tinha 210 leitos, recebeu 3.237 pacientes com COVID-19 suspeita ou confirmada. Na análise dos primeiros 616 pacientes admitidos, percebemos que nossa epidemiologia foi semelhante aos dados descritos internacionalmente, ^( 2 )^ exceto para uma população relativamente mais jovem: 57,4% eram homens, com mediana de idade de 55 anos e variação de zero a 95 anos. As comorbidades mais comuns foram hipertensão (53,6%), diabetes (35,7%) e obesidade (24%). Embora menos de 10% da amostra do estudo tinha doenças respiratórias crônicas conhecidas, um quarto dos pacientes era ex-fumante ou fumante atual. A duração mediana dos sintomas antes da admissão hospitalar foi de 7 dias, e a maioria dos pacientes apresentou febre (72,2%), tosse (79,6%) e dispneia (78,3%). Mialgia e fadiga também foram queixas comuns.

Na apresentação, quase 70% necessitavam de algum tipo de suplementação de oxigênio ou tinham infiltrados pulmonares que ocupavam mais de 50% do parênquima, sendo considerados como tendo doença grave. Embora o tempo desde o início dos sintomas não fosse diferente entre pacientes com doença grave ou leve, aqueles com doença grave eram mais velhos, com mais comorbidades (especialmente diabetes, hipertensão e outras doenças cardiovasculares crônicas) e apresentavam relação menor entre pressão parcial de oxigênio/fração inspirada de oxigênio (PaO _2_ /FiO _2_ ). A suplementação inicial de oxigênio foi feita por cânulas nasais (60,4%) ou máscaras de reservatório não reinalantes (17,8%) para a maioria dos casos graves, mas 9,6% necessitaram de ventilação mecânica invasiva imediata.

Em geral, conseguimos obter resultados clínicos semelhantes aos relatados nos Estados Unidos e em países europeus, ^( 3 )^ com mortalidade intra-hospitalar de 24,5%. Como esperado, aqueles com doença leve tiveram hospitalização mais curta (mediana de 3 *versus* 7 dias), alta mais rapidamente (64,2% *versus* 37%) e mortalidade hospitalar mais baixa (6,6% *versus* 32,8%) do que os que apresentavam doença grave na admissão. Ademais, os pacientes com doença grave necessitaram de cuidados intensivos com mais frequência (39,8% *versus* 8,1%), ventilação mecânica (39,9% *versus* 9,8%), vasopressores (30,9% *versus* 8,1%) e bloqueio neuromuscular (27% *versus* 4,9%) em algum momento da hospitalização. Além disso, a incidência de lesão renal aguda foi maior entre pacientes com doença grave (35% *versus* 12,5%), assim como a necessidade de terapia renal substitutiva (17,9% *versus* 2,5%). É importante enfatizar que todos esses números refletem a realidade antes dos resultados do estudo RECOVERY, ^( 4 )^ de forma que corticoesteroides foram administrados apenas a uma pequena fração de pacientes incluídos em estudos clínicos.

Como um hospital comunitário de Atenção Secundária, que, historicamente, esforçava-se para conseguir sobreviver, alcançou resultados comparáveis aos de centros americanos e europeus? Distinguir crise de desastre é o primeiro passo para criar um senso de urgência. Crise requer decisão e preparação. Desastre, por sua vez, é um evento imprevisível e fora de qualquer controle. Confiando na experiência internacional e analisando com frequência seu próprio conjunto de dados, o HMMD foi capaz de antecipar e preparar a instituição para a crise que viria, aprendendo e compartilhando experiências com outras instituições médicas do país e do exterior.

A alocação racional de recursos e com boa relação custo-efetividade foi fundamental para uma resposta assertiva à crise que chegava. ^( 5 )^ Como ilustrado pelas estatísticas aqui apresentadas, tanto os recursos humanos quanto os tecnológicos tiveram que ser rapidamente aumentados para atender à crescente demanda de casos de pacientes complexos e críticos. Em um nível sem precedentes de colaboração com o HIAE e a indústria privada, em questão de semanas o HMMD recebeu 190 ventiladores mecânicos, 60 monitores multiparamétricos e 30 máquinas de hemodiálise. Além disso, várias áreas do hospital, como enfermarias médicas e cirúrgicas, centros cirúrgicos e ambulatórios, foram rapidamente adaptadas para receber pacientes críticos. De modo impressionante, um novo hospital anexo, com capacidade para cem pacientes, foi construído em apenas 23 dias. O resultado foi um processo de escalada extremamente rápido por meio do qual o hospital comunitário de Atenção Secundária, de médio porte, foi essencialmente transmutado em um grande centro de referência, com capacidade para 524 pacientes e 220 leitos de cuidados intensivos, em menos de 1 mês.

Em termos de recursos humanos, foram abertas 1.080 novas vagas para médicos e outros profissionais de saúde. Treinamento contínuo e comunicação utilizando ferramentas de simulação e videoconferência foram fundamentais para manter a equipe engajada e atualizada sobre a conduta na COVID-19. A crise trouxe de volta um profundo senso de propósito e trabalho em equipe para muitos profissionais de saúde. O propósito – e não o salário – foi o que levou os colegas a virem trabalhar conosco. Foi um chamado para fazer a diferença e criar um legado, independentemente dos plantões extras e da exaustão. Curiosa e felizmente, conseguimos transmitir esse mesmo sentido de propósito aos nossos estudantes de medicina e residentes, que continuaram com seu treinamento e contribuíram de forma significativa no cuidado de pacientes.

À medida que os índices de infecção diminuirem e emergimos da pandemia da COVID-19, é natural que olhemos para trás e meditemos sobre isso. Desde o dia desta redação, 554 vidas foram perdidas somente em nosso hospital desde março. Eram pais, avós, filhos e filhas de outras pessoas. Porém enquanto sua perda irreparável provocou repetidas revisões de nossos processos e diretrizes institucionais, os 2.181 pacientes que foram tratados com sucesso e receberam alta são a prova viva das três lições mais importantes desta pandemia: primeiro, podemos fazer milagres quando os setores privado e público se unem para o melhor interesse das pessoas; segundo, o trabalho colaborativo é a melhor maneira de construir conhecimento e salvar vidas; e terceiro, de um ponto de vista individual, podemos nunca mais experimentar um crescimento pessoal tão grande em nossa vida.
